# Reduction of Inflammation and Colon Injury by a Spearmint Phenolic Extract in Experimental Bowel Disease in Mice

**DOI:** 10.3390/medicines6020065

**Published:** 2019-06-06

**Authors:** Rosa Direito, João Rocha, Ana Lima, Maria Margarida Gonçalves, Maria Paula Duarte, Vanessa Mateus, Catarina Sousa, Adelaide Fernandes, Rui Pinto, Ricardo Boavida Ferreira, Bruno Sepodes, Maria-Eduardo Figueira

**Affiliations:** 1 Research Institute for Medicines and Pharmaceutical Sciences (iMed.UL), Faculty of Pharmacy, Universidade de Lisboa, Av. Prof. Gama Pinto, 1649-003 Lisbon, Portugal; rosadireito2008@gmail.com (R.D.); jrocha@ff.ulisboa.pt (J.R.); vanessa.mateus@estesl.ipl.pt (V.M.); sousacatarina@campus.ul.pt (C.S.); amaf@ff.ulisboa.pt (A.F.); rapinto@ff.ulisboa.pt (R.P.); bsepodes@ff.ulisboa.pt (B.S.); 2 Disease & Stress Biology Group, LEAF, Instituto Superior de Agronomia, Universidade de Lisboa, 1349-017 Lisbon, Portugal; agusmaolima@gmail.com (A.L.); rbferreira@isa.utl.pt (R.B.F.); 3 Unidade de Biotecnologia Ambiental, Universidade Nova de Lisboa, Quinta da Torre, 2829-516 Monte da Caparica, Portugal; mmpg@fct.unl.pt; 4 Unidade de Biotecnologia Ambiental (UBiA), Grupo de Disciplinas da Ecologia da Hidrosfera, Faculdade de Ciências e Tecnologia, FCT, Universidade Nova de Lisboa, 2829-516 Caparica, Portugal; mpcd@fct.unl.pt; 5 H&TRC–Health and Technology Research Center, ESTeSL–Lisbon School of Health Technology, Instituto Politécnico de Lisboa, 1990-096 Lisbon, Portugal; 6 Joaquim Chaves Saúde, Dr Joaquim Chaves Lab Analises Clínicas, 1495-068 Miraflores-Algés, Portugal

**Keywords:** spearmint, *Mentha spicata* L., phenolics, inflammation, colitis, colorectal cancer

## Abstract

**Background**: Inflammatory Bowel Diseases (IBD) encompasses both Crohn’s Disease and Ulcerative Colitis, known to be connected to an enlarged risk for developing colorectal cancer (CRC). Spearmint (*Mentha spicata* L.) is a Mediterranean plant used as an aromatic agent, and studies have mainly focused on the essential oil suggesting an anti-inflammatory activity. This work aimed to perform a preliminary screening of the in vivo anti-inflammatory effects of a spearmint phenolic extract in an acute inflammation model, in a chronic inflammation model of colitis, and also study the effects in vitro on a colon cancer model. **Methods**: Spearmint extract was administered to rats of a paw oedema model (induced by carrageenan) and to mice from a TNBS-induced colitis model in parallel with studies using HT-29 CRC cells. **Results**: Administration of the extract led to reduced paw inflammation, reduction of colon injury and inflammation, with attenuation of histological markers, and reduction of iNOS expression. It repressed the in vitro movement of HT-29 cells in a wound healing assay. **Conclusions**: These findings suggest that spearmint extract exhibits acute and chronic anti-inflammatory activity and is able to inhibit migration of cancer cells, suggesting a potential role in the supplementary therapy of IBD patients.

## 1. Introduction

The Inflammatory Bowel Disease (IBD) condition is a chronic and incapacitating disease, which is classified into two archetypal phenotypes, ulcerative colitis (UC) and Crohn’s disease (CD). Nowadays it affects millions of patients in Europe and in the USA, with a rising number of cases in many nations in South America, the Middle East and Asia, attributed to the adoption of a “westernized style” of life [1,2,3,4]. IBD is characterized by intestinal symptoms such as abdominal pain, bloody diarrhoea, rectal urgency and tenesmus [5]. Since there is excessive proliferation required to repair the intestinal tissue injury, IBD is associated to an augmented risk of developing colorectal cancer (CRC) parallel to the common population (20- to 30-fold risk). The risk of developing CRC also increases significantly 8–10 years after diagnosis of IBD. It is also known that CRC associated with IBD is more severe and associated to a higher mortality rate than CRC without this risk factor [6,7,8]. Colonic carcinogenesis exemplifies the connection between chronic inflammation and the origins of cancer and has long been investigated. Colitis-associated colorectal cancer (CAC) is a tumour that develops within the context of chronic inflammation, and is considered the most serious complication of IBD [8].

Spearmint (*Mentha spicata* L.), usually used as a flavouring agent, has demonstrated both antioxidant and anti-inflammatory effects in chronic obstructive pulmonary disease (COPD) and irritable bowel syndrome [9,10]. Spearmint contains a wide variety of active compounds, with rosmarinic acid (RA) being one of the most significant ones [11,12]. RA has shown evidence of exhibiting antioxidant, anti-inflammatory, antibacterial, antiviral, neuroprotective, hepatoprotective and immunosuppressant activities [13,14,15,16,17,18]. Tumour cells are known to have specific populations of neutrophils, known as tumour-associated neutrophils (TANs) that possess tumour-supportive functions. Some studies have demonstrated that TANs promote the spread of cancer cells to distant organs by contributing to tumour invasion and angiogenesis through the production of matrix metalloproteinase-9, vascular endothelial growth factor (VEGF), and hepatocyte growth factor (HGF) in the primary and metastatic sites [19].

Current pharmacological management of IBD includes corticosteroids, aminosalicylates, immunosuppressants and biological agents. Currently, the aim of therapy is only to bring and/or keep the patient in remission and improve the symptoms associated with the disease, rather than modifying or reversing the underlying pathogenic mechanism. It is acknowledged that drug therapy in IBD could result in significant adverse effects [5,20]. Thus, the research for new pharmacological approaches that aims both at remission and maintenance with fewer side-effects is critical to promote a most wanted significant advancement in the pharmacological management of IBD, contributing to a more effective and selective treatment compared to what is currently known. Additionally, any add-on therapy that helps to reduce the burden of existing therapeutic options while potentiating an anti-inflammatory therapeutic effect would be strongly beneficial for patients. In this regard, supplementation with natural products might offer an interesting option.

Although spearmint has been extensively studied, the available studies have only focused primarily on the essential oil, and not on the plant as a whole. The leaf has more compounds besides the ones that are present in the essential oil, which may have a synergic effect. Considering that chronic inflammation results from unresolved acute inflammation processes, and cancer from the perpetuation of persistent and prolonged chronic inflammation processes over time, the more time the inflammation persists, the higher the risk of associated carcinogenesis [21,22].

The goal of this work was to characterise the chemical composition of a spearmint leaf extract (SE) and to evaluate its acute and chronic anti-inflammatory and anti-oxidant assets that are relevant for a pharmacological intervention in IBD and to prevent the development of CAC.

In this study we performed a paw oedema assay (acute inflammation model) and a TNBS-induced colitis model (chronic inflammation model) to add to the study of the anti-inflammatory properties of the SE, and the anti-proliferative effect of the extract using an in vitro HT-29 human colon adenocarcinoma cells was additionally tested.

## 2. Materials and Methods 

### 2.1. Drugs and Chemicals

Xilazine (Rompun^®^ 2%) and ketamine (Imalgene^®^ 1000) were acquired from Bio2 *Produtos Veterinários* (Lisboa, Portugal). Unless otherwise stated, all remaining substances were purchased from Sigma-Aldrich, Lisboa, Portugal. 

### 2.2. Preparation of the Spearmint Extract

Spearmint leaves (*Mentha spicata* L.) sourced from local Portuguese plantations were used in this study. Spearmint fresh leaves (30 g) were first washed and left to dry in the dark at room temperature. After drying, these spearmint leaves were crushed and the extract was prepared by adding 100 mL of ethanol:water (70:30, *v*/*v*) to the remaining 20 g of spearmint leaves, which were magnetically agitated for 24 h. The extract was then filtrated under vacuum with filter paper Whatman no.41; 0.45 µm, and the filtrate was washed with ethanol 70% in the proportion of 30 mL of extract to 100 mL of ethanol. To eliminate the ethanol, the resulting extract was submitted to evaporation in a rotative evaporator at 40 °C and then centrifuged (5000 rpm; 15 min; 4 °C). The total supernatant was collected and re-filtered, and subsequently evaporated at 40 °C to almost dryness and then centrifuged again under the same conditions. Finally, the spearmint was filtered to prevent microorganism growth. The concentrated spearmint (60 mL) was kept at 4 °C and in the dark, prior to study application.

### 2.3. Phenolic Characterization of Spearmint Extract

The phenolic components of the aqueous extract were purified by adsorption to solid phase extraction C18 columns (500 mg/3 mL, Ref. 7020-03, J.T. Baker, Porto Salvo, Lisboa, Portugal) and the mobile phase used was acetonitrile and formic acid in water (0.1% *v*/*v*). The eluted extracts were combined and concentrated to their initial volume and clarified through a 0.22 µm filter previously examined. The chromatographic examination was performed in an HPLC system (SpectraSystem, Thermo, Darmstadt, Germany), furnished with a Thermo C-18 column, and a diode array detector (DAD). Elution was performed with 0.1% formic acid (solvent A) and a mixture of 90% acetonitrile + 9.9% water + 0.1% formic acid (solvent B), at a stream speed of 0.8 mL/min. The pump was programmed with 2% solvent B during 10 min, increasing to 20% at 60 min and to 98% at 140 min, followed by a re-equilibration step of 10 min to reach the initial condition of 2% B. Spectra acquisition was made in the range of 190 nm to 700 nm with selective detection at 280 nm (phenolics in general, hydroxybenzoic acids, procyanidins or catechins), 320 nm (hydroxycinnamic acids, flavones) and 360 nm (flavonols). Identification of the main functional groups present in the extract was performed by a comparison of their UV spectra with those of representative standards analysed under the same conditions and from literature data. The determination of Total phenolic compounds (PCs) concentration present in the spearmint was assessed by the Folin–Ciocalteu method [23]. Gallic acid was applied as standard, and the assay results were expressed as means of at least three replicates (mg gallic acid equivalents (GAE)/L and mg GAE/g of spearmint). RA quantification was performed by response factor calculations using the chromatographic area of a 1 g/L standard co-injected in the same analytical conditions.

### 2.4. Determination of the Antioxidant Activity

Spearmint’s antioxidant activity was determined by the Ferric Reducing Antioxidant Power (FRAP) method reducing the activity of Fe(III) to Fe(II) according to the Ramful method [24]. The results were expressed as means of three replicates (μmol Fe^2+^/mL of spearmint and μmol Fe^2+^/g of dried leaves).

The determination of Cupric Reducing Antioxidant Capacity (CUPRAC) agreed with Apak methodology [25]. The results were expressed as means of three replicates (μmol ascorbic acid equiv (AAE)/mL of spearmint and μmol AAE/g of dried leaves).

The 2,2-diphenyl-1-picrylhydrazyl (DPPH) radical-scavenging activity was measured in agreement with Miceli methodology [26]. The result was expressed as means of three replicates (mg AAE/mL of spearmint and mg AAE/g of dried leaves).

The spearmint capacity for scavenging the superoxide anion radical was evaluated in agreement with the methodology reported in [27]. Analyses were performed in triplicate and results were presented as means of three replicates (μmol GAE/mL of spearmint and μmol GAE/g of dried leaves).

### 2.5. Animals for the in vivo Experimental Models

Male rats from Wistar strain, with body weight between 100–150 g and five weeks old (Harlan Iberica, Barcelona, Spain) were used to perform the carrageenan-induced paw oedema assay. 

Male CD-1 mice with 30–40 g body weight and 6–10 weeks old (Charles River, Barcelona, Spain) were employed to perform the TNBS-induced colitis model. Animals were accommodated in polypropylene cages with free access to water and food and kept at a temperature of 18–23°C and 40%–60% of humidity in a light/dark cycle of 12 h at the Faculty of Pharmacy (University of Lisbon, Lisbon, Portugal). Experiments were performed agreeing to the most recent rules and recommendations for the care and processing of laboratory animals, namely to the presently adopted European Commission regulations (Directive 2010/63/EU). In addition, the studies were performed in agreement with the ARRIVE Guidelines for Reporting Animal Research. The Ethics Committee of the Faculty of Pharmacy (University of Lisbon) also endorsed the experimental protocol (0019/2018; Date of approval: 27 February 2018).

### 2.6. Acute Imflammation Rodent Model Assay

The paw oedema in the rat’s left hind paw was induced by intradermal (sub-plantar) injection of 100 µL of a λ-carrageenan (1% in saline) as previously described [18]. Paw volume measurements were the following: V0 or basal volume is the hind paw volume measured immediately after carrageenan injection plus V6 corresponding to volume at 6h post carrageenan administration. The rise in paw volume was measured as the oedema volume and was expressed as a relative percentage, calculated following the formula:

$$\%\;\mathrm{paw}\;\mathrm{volume}\;\mathrm{increase}=\frac{\left(V6-V0\right)}{V0}\times 100$$

Animals remained randomized within six experimental groups:

- Control group (n = 8): They were sub plantar injected in their left hind paw with 0.1 mL sterile saline and orally administered with water (1 mL/kg) by gavage, used as spearmint vehicle, 30 min before carrageenan injection.
- Carrageenan group (n = 8): Group was subjected to paw oedema induction by injection into their left hind paw of 0.1 mL of carrageenan (1%) and orally administered with water (1 mL/kg) by gavage, used as spearmint vehicle, 30 min before κ-carrageenan injection.
- Spearmint group (n = 6): Rats exposed to paw oedema induction and pre-treated with spearmint extract (15 mg/kg) by oral gavage, 30 min prior to carrageenan injection.
- Indomethacin group (n = 6): Rats were exposed to paw oedema induction and pre-treated with indomethacin (10 mg/kg) by oral gavage, used as a positive control, 30 min prior to carrageenan injection.
- Trolox group (n = 6): Rats exposed to paw oedema stimulation and pre-treated with trolox (10 mg/kg) by oral gavage, used as a positive control, 30 min prior to carrageenan injection.
- Tempol group (n = 6): Rats exposed to paw oedema stimulation and pre-treated with tempol (10 mg/kg) by oral gavage, used as a positive control, 30 min before carrageenan injection.

### 2.7. Induction of Rodent Colitis Model

The induction of colitis was accomplished by administration of 100 µL of 2.5% (*w*/*v*) TNBS (50% ethanolic solution by intracolonic administration) as previously described [28]. Four experimental groups of animals were designed with random distribution:

- Sham group (n = 4): The colitis induction protocol was followed as described above with the exception for intracolonic administration which was with 100 μL of saline solution instead of the alcoholic TNBS solution. Water was administered to animals (10 mL/kg) by oral gavage throughout the four days of the experiment.
- Ethanol group (n = 4): The colitis induction protocol was followed as described above with the exception for the intracolonic administration that was with 100 μL of 50% (*v*/*v*) ethanol solution instead of the alcoholic TNBS solution. Water was administered to animals (10 mL/kg) by oral gavage throughout the four days of the experiment.
- TNBS group (n = 8): The colitis induction protocol was followed as described above, with the administration of 100 μL of TNBS. Water was administered to animals (10 mL/kg) by oral gavage throughout the four days of the experiment.
- TNBS + Spearmint group (n = 9): The colitis induction protocol was followed as described in the previous experimental group. Spearmint extract (15 mg/kg of phenolic acids by oral gavage) was administered to animals throughout the four days of the experiment.

On the 4th day post-induction, cardiac puncture under surgical anaesthesia was performed and blood samples were collected, followed by euthanasia by cervical disarticulation and subsequent necropsy. The colon was collected and was observed for cataloguing of diarrhoea harshness. Furthermore, the colon was washed with PBS for a macroscopic evaluation of lesions of the colon tissue wall (e.g., erosion, perforation, necrosis) and fixed in 4% of PFA in PBS for histological studies. Diarrhoea severity (proxy to macroscopic evaluation of colitis severity) was classified by a technician blind to the experimental groups agreeing to Table 1. A microscope observation of the tissue was performed followed by measurement of the entire colon and injury extent.

### 2.8. Histologic and Immunohistochemistry Evaluation

The histologic Haematoxylin & Eosin (H&E) staining was done as previously reported [28], as well as the immunohistochemistry studies, for measurement of cyclooxygenase-2 (COX-2) and inducible nitric oxide synthase (iNOS) expression. 

The tissues were fixed in 4% PFA in PBS for 72 h at room temperature, decalcified, dehydrated in a series of graduated ethanol and embedded in paraffin. Immunostaining was done in sections cut to a thickness of 6 mm and subjected to antigen retrieval in 20 mM citrate buffer with 1.5% H_2_O_2_ for 15 min at room temperature in the dark, incubated for 10 min in Tris buffer/EDTA at 84 °C and blocked for 1 h at room temperature in 1% bovine serum albumin (BSA) in PBS; following the application of the primary, rabbit anti-COX-2 and rat anti-iNOS antibodies in 0.5% BSA in PBS overnight at 4 °C. After washing in PBS, the sections were incubated for 1 hour at room temperature with rabbit anti-rabbit and anti-mouse antibodies to horseradish peroxidase in 0.5% BSA in PBS and incubated for 10 min in SIGMAFAST ™ DAB assembled with EntellanÒ. These tissue samples were analysed on an Axioskop field light microscope and images were obtained with a DFC 490 (Leica) camera, which transformed them into inverted images and 8-bit files. The intensity of protein staining corresponds to the level of expression of COX-2 and iNOS. The level of iNOS or COX2 staining was quantitatively evaluated by determining the percentage of tissue area that was stained with brown staining, using ImageJ (Fiji Is Just) software, version 1.47 (National Institutes of Health (NIH), Bethesda, MD, USA).

The severity of inflammation and colon injury was graded semi-quantitatively according to the following scale: 0–3: normal colon with no lesions, mucosa of same thickness, straight crypts, normal crypt structural design, no cellular infiltration, oedema or exudate significance with no marks of inflammation; 1: colon with slight lesions, mucosal erosion with small superficial ulcers dispersed along the extent of the colon, with small crypt loss and neutrophil infiltration; 2: colon with restrained lesions, bowels with extensive erosion and ulceration, with moderate crypt loss and neutrophil infiltration; 3: colon with very austere ulceration, slim mucosa with loss of crypts and noticeably increased infiltration of neutrophils and severe inflammatory exudate.

### 2.9. HT-29 Cells Experimental Assays

The cell line ECACC, number 91072201, the HT-29 colon adenocarcinoma cells from *Homo sapiens sapiens*, was used in in vitro experiments, as previously described [29]. The assays performed were:

- Wound healing assay: The spearmint concentration of 500 µg phenolics/mL was further evaluated for its inhibitory activity on HT-29 colon cancer cells. For cell migration study, the wound healing assay was achieved as previously reported [30]. The counting of migrating cells into the wound gap, in three random fields, from each triplicate treatment was performed, and data are expressed as the mean ±S.D.
- Matrix metalloproteinase-9 (MMP-9) catalytic activities: MMP-9 catalytic activities were performed as previously described [28]. The fluorescence at ex. 485 nm/em. 530 nm was measured.
- Minimal Inhibitory Concentration (MIC) of extract on catalytic activity of MMP-9 inhibition was assessed using Minimal Inhibitory Concentrations (MICs) that were evaluated using the micro-dilution process as previously described in ref. [30].
- The DQ gelatine assay was assessed as reported in ref. [30] with the following changes: SDS-polyacrylamide gels (12.5% *w*/*v* acrylamide) were copolymerized with 1% (*w*/*v*) gelatine. Cell culture supernatants treated with a non-reducing buffer (62.6 mM Tris–HCl pH 6.8), 10% (*v*/*v*) glycerol, 2% (*w*/*v*) SDS and 0.01% (*w*/*v*) bromophenol blue were loaded into each well of the SDS-gel. Electrophoresis was carried out as described before [30] in a 12% (*w*/*v*) acrylamide resolving gel and a 4% (*w*/*v*) acrylamide stacking gel, did in a vertical electrophoresis unit at 100 V and 20 mA per gel. After electrophoresis, gels were washed three times using 2.5% (*v*/*v*) Triton X-100 for 90 min each, to remove the SDS. Gels were then incubated overnight with developing buffer (50 mM Tris–HCl pH 7.4, 1 μM ZnCl2, 5 mM CaCl_2_ and 0.01% *w*/*v* sodium azide), stained with Coomassie Brilliant Blue G-250 0.5% (*w*/*v*) in 50% (*v*/*v*) methanol and 10% (*v*/*v*)acetic acid, for 30 min, and de-stained with a solution of 10% (*v*/*v*) acetic acid, 50% (*v*/*v*) methanol. White bands visible against a blue background marked the gelatinase activity of each proteinase.

### 2.10. Statistical Analysis

All data is communicated as mean ± S.D. One-factorial ANOVA test was applied to all results to compare these. A Bonferroni’s post hoc test was performed afterwards. The software GraphPad Prism 5.0 (GraphPad, San Diego, CA, USA) was used. A Kaplan–Meyer analysis was performed to assess the differences in the onset of mortality, followed by a Mantel–Cox Test. *p*-values lower than 0.05 were considered to be statistically significant. 

In the in vitro assay with HT-29 cells and gelatinolytic activities, all tests were performed in triplicate, at least three different times, with the results being communicated as the mean ± S.D. SigmaPlot software (version 12.5, Systat Software Inc. (SSI), San Jose, CA, USA) was applied so as to match different handlings, using one-way or two-way ANOVA. To compare differences between groups Tukey’s test was used, as well as Dunnett’s test. *p*-values lower than 0.05 were considered to be of statistical significance.

## 3. Results

### 3.1. Phenolic Composition of Spearmint Extract

**Figure 1:**
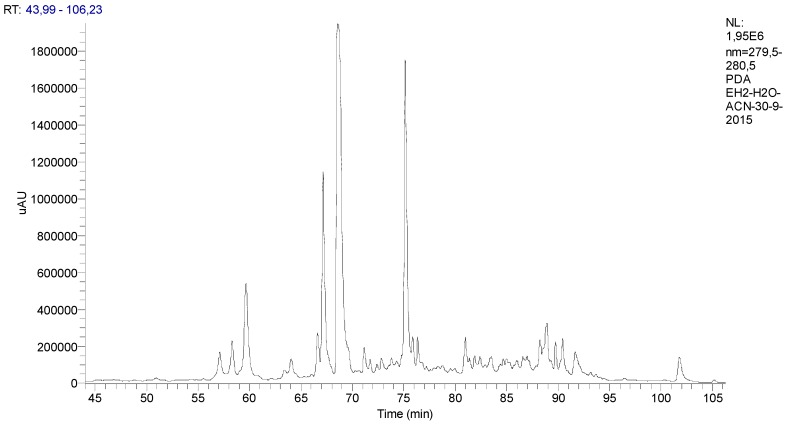
Chromatographic profile of the *Mentha spicata* L. extract with identification of its phenolic components classified according to their functional groups.

The chromatographic profile of the spearmint is presented in Figure 1.

**Figure 2:**
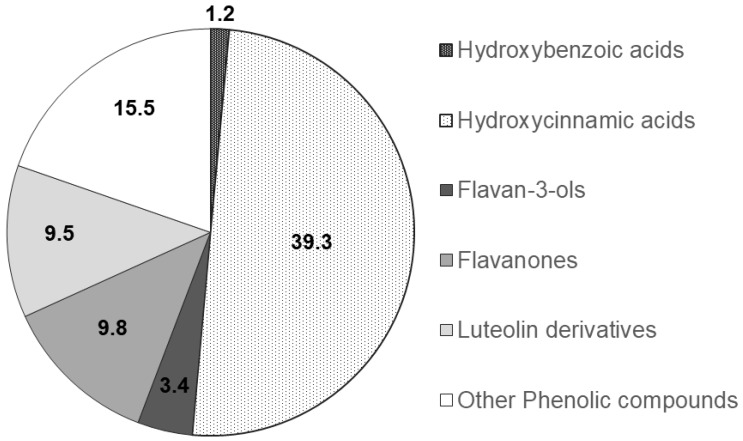
Relative chromatographic area distribution of the main phenolic functional groups detected in the *Mentha spicata* aqueous extract.

The functional groups of the main PCs were recognised through a comparison of the corresponding spectra available within literature data and their relative chromatographic areas (r.c.a.) are presented in Figure 2.

### 3.2. Determination of the Antioxidant Activity

Considering the requirement for performing multiple assays to corroborate the extract’s antioxidant effect, the team ran FRAP, CUPRAC, superoxide anion radical scavenging assay and DPPH radical-scavenging, to expand the understanding of the likely machinery of the spearmint. The results are presented in Table 2. 

### 3.3. Acute Inflammation Rodent Model Assay

**Figure 3:**
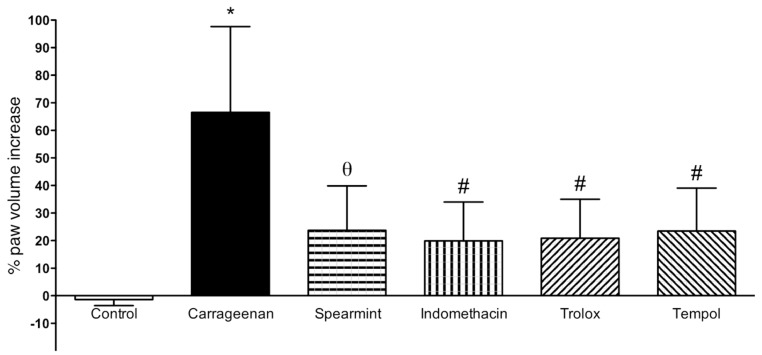
Effect of Spearmint extract administration on the paw oedema volume induced by carrageenan. * *p* < 0.001 vs. Control; θ *p* < 0.01 vs. Carrageenan; # *p* < 0.001 vs. Carrageenan.

The results from paw oedema analysis are presented in Figure 3. 

In comparison to the sham group, sub plantar instillation of carrageenan in rats led to a noteworthy increase of paw volume after 6 h (60 ± 20%). Paw oedema volume in the group treated with spearmint was reduced by 36% (24 ± 16%; *p* < 0.01), when compared to the carrageenan group. Regarding the positive controls (indomethacin, trolox and tempol) their results show a similar effect comparing with spearmint.

### 3.4. Macroscopic Evaluation of Colitis Severity

The results obtained from the macroscopic evaluation of colitis severity, like colon length, diarrhoea and lesion extension scores are represented in Table 3, as well as the mortality rates. 

**Figure 4:**
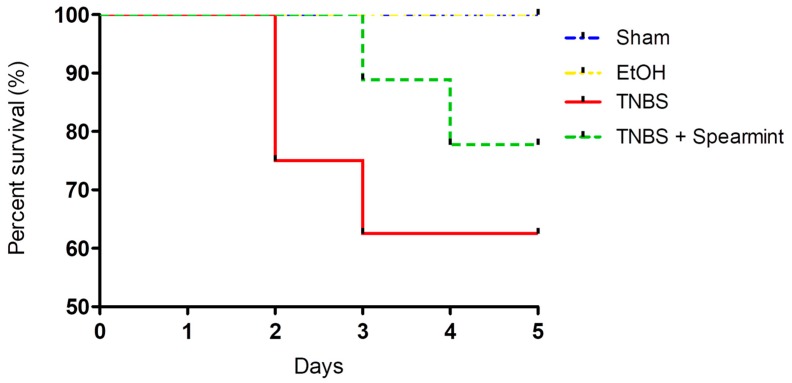
Comparison of survival curves between the four groups tested in the in vivo colitis model.

The differences between groups in the onset of mortality were analysed (Figure 4).

This analysis revealed that survival curves in the four groups tested did not have statistically significant differences.

**Figure 5:**
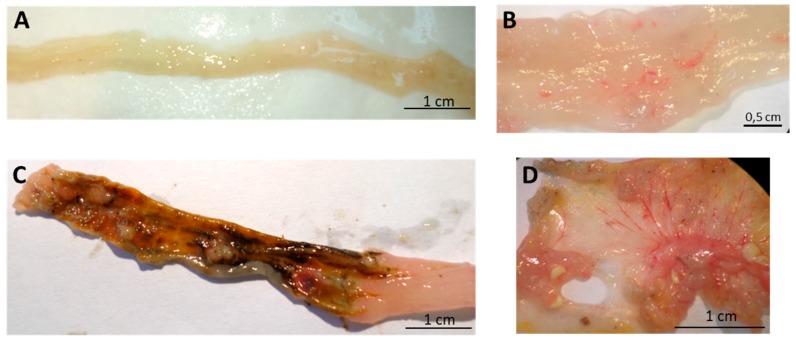
Effect of oral administration of spearmint extract, on the macroscopic evaluation of colon. (**A**) Sham group (n = 4), (**B**) EtOH group (n = 4), (**C**) TNBS group (n = 8), (**D**) TNBS + Spearmint extract group (n = 9).

**Figure 6:**
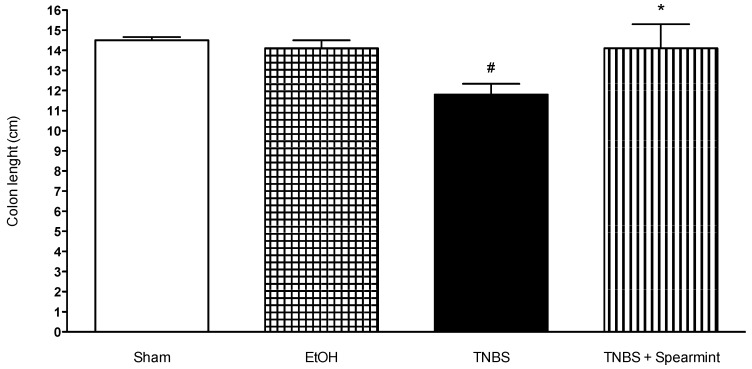
Effect of spearmint extract administration on colon length (cm). # *p* < 0.001 vs. Sham; * *p* < 0.001 vs. TNBS.

**Figure 7:**
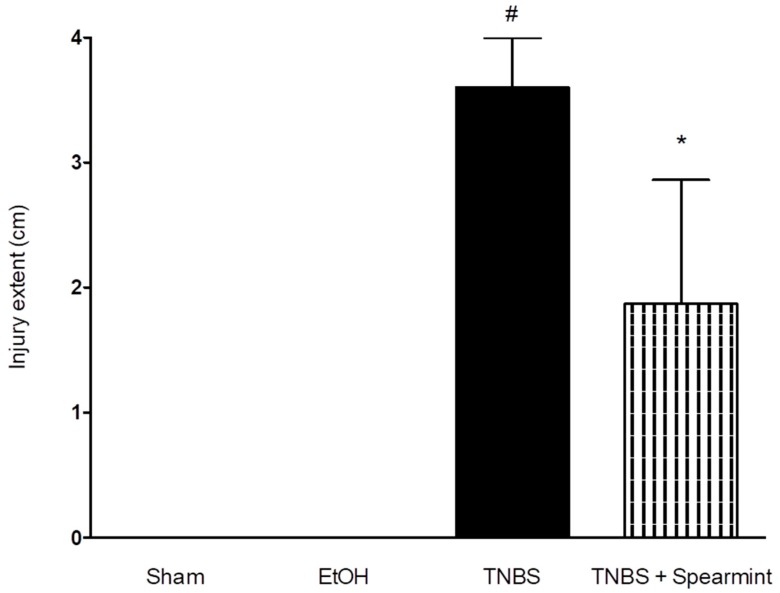
Effect of oral administration, of the spearmint extract on the extent of intestine injury (cm). # *p* < 0.001 vs. Sham; * *p* < 0.001 vs. TNBS.

Images of the macroscopic colon lesions were also analysed in Figure 5, Figure 6 and Figure 7.

The TNBS + Spearmint group presented some lesions but they were shorter and less severe when compared to the TNBS group (Figure 7). The diarrhoea score was close to 1 and the colon length did not exhibit any significant reduction when compared to TNBS mice (Figure 6). Also, the mortality rate of TNBS + Spearmint group decreased to 22.2% when related with the TNBS group.

### 3.5. Histologic and Immunohistochemistry Evaluation

**Figure 8:**
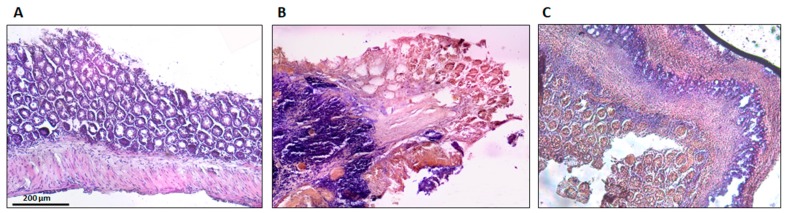
Effect of the spearmint extract administration on the histological features of colon inflammation. (**A**) Sham group, (**B**) TNBS group, (**C**) TNBS + spearmint group. Original magnification × 100. Scale bar equals 200 µm.

The histological findings are shown in Figure 8. Sham group colons were normal, without lesions, with uniform mucosa thickness and normal crypt structural design; inflammation signs were also not identified. The TNBS group presented critical lesions with ulcerations, crypt loss and a thinner thickness of mucosa; also presented significant signs of inflammation with neutrophilic infiltration, equivalent to a score of 3. The TNBS + Spearmint group presented less severe lesions and ulcers. Most of the lesions were small superficial ulcers that correspond to a score of 1 with some of those presenting a score of 2. The crypt loss was minimal, and the inflammation signs were also minimal with some neutrophilic infiltration.

**Figure 9:**
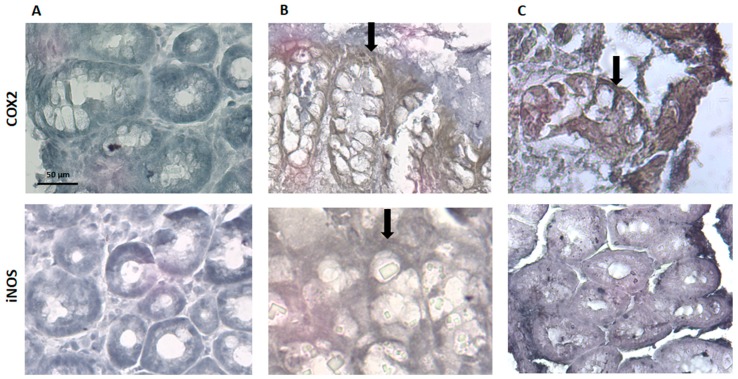
Effect of spearmint phenolic extract administration on the colon tissue expression of COX-2 and iNOS. The production of COX-2 and iNOS are exhibited by brown staining and arrows (**A**) Sham group, (**B**) TNBS group, (**C**) TNBS + spearmint group. Original magnification × 100. Scale bar equals 50 µm.

Regarding COX-2 and iNOS expression, they are identified by a ‘brownish’ coloration (Figure 9). The sham group did not present any brown coloration, which indicates that the expression of COX-2 and iNOS, if any, appears to be insignificant, and this is consistent with the absence of an inflammatory stimulus. The TNBS group showed an increased expression of COX-2 and iNOS, which is in accordance with the previous studies, given the inflammation associated to colitis in these animals [28]. The spearmint group presented a reduced expression of iNOS although the COX-2 expression was not significantly inhibited (Figure 9C).

**Figure 10:**
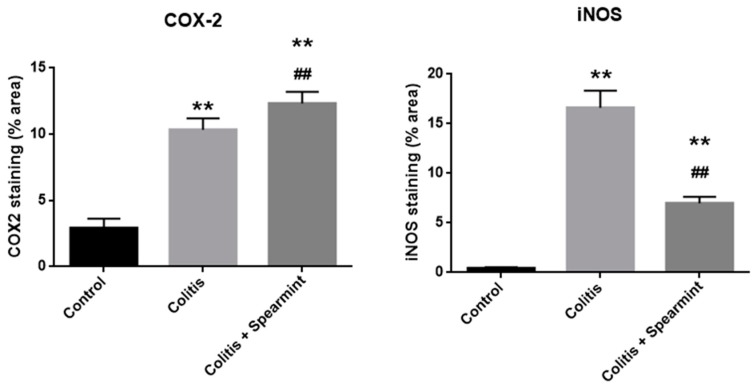
Effect of spearmint phenolic extract administration on the colon tissue expression of COX-2 and iNOS. The production of COX-2 and iNOS staining were quantified. ** *p* < 0.01 vs. Control; ## *p* < 0.01 vs. Colitis.

The evaluation of the iNOS and COX-2 staining were quantified (Figure 10), and the results show the reduction in iNOS expression in the presence of spearmint extract and almost no inhibition of COX-2 expression.

### 3.6. HT-29 Assays

**Figure 11:**
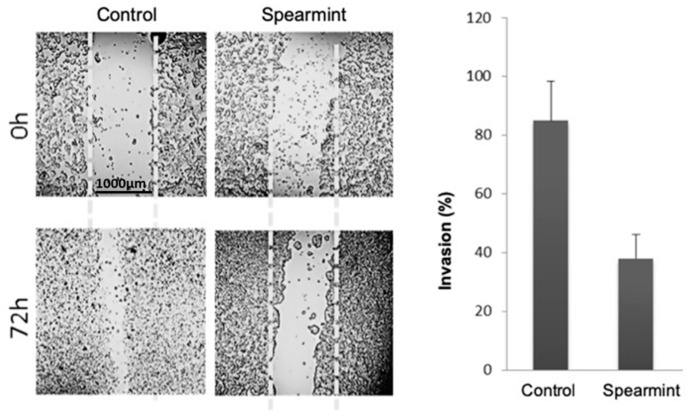
Effect of spearmint phenolic extract (at a concentration of 500 μg/mL) on the invasion properties of HT-29 colorectal cancer cells in a wound-closure assay (at 0 h and 72 h).

Given the importance of inflammatory signalling in colitis progression into colorectal cancer, we aimed to evaluate if the anti-inflammatory effects exhibited by the spearmint extract in the animal models of acute and chronic inflammation would be able to disrupt the tumorigenesis capacity of human colon adenocarcinoma cancer cells line (HT-29 cells), through the evaluation of migration/invasion properties (responsible for tumour invasion and metastasis capabilities) and their relation to MMP expression and activity. Using the wound-closure assay, in which cells depend on their migration and invasion properties to fill an induced empty space in the cell layer, spearmint extract incubation with HT-29 cells inhibited cell invasion by more than 50% when compared to control samples (Figure 11).

**Figure 12:**
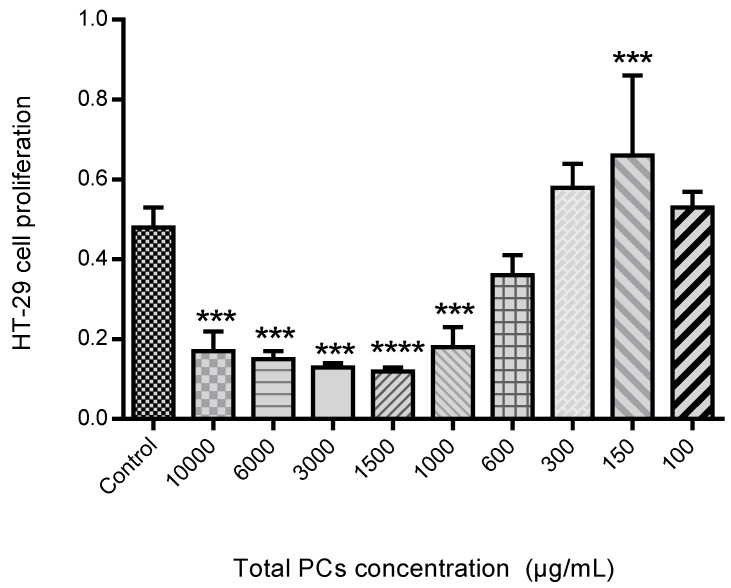
Effect of spearmint phenolic extract, at different concentrations of PCs, on the HT-29 cells proliferation. *** *p* < 0.001 vs. Control; **** *p* ˂0.0001 vs. Control.

The proliferation assay was performed as can be seen in Figure 12. The HT-29 cells proliferation in the presence of different PCs concentration, exhibited that spearmint extract inhibited cell proliferation by more than 50% at concentrations less than 600 μg/mL, when compared to control samples and the differences among means of each group were statistically significant.

**Figure 13:**
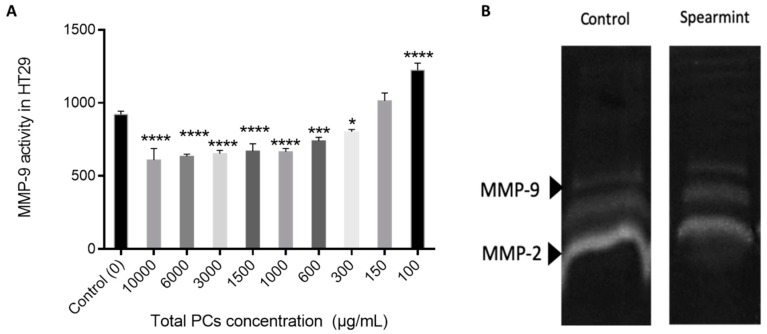
(**A**) Effect of spearmint phenolic extract on the MMP-9 activity. * *p* < 0.05 vs. Control; *** *p* < 0.001 vs. Control; **** *p* ˂ 0.0001 vs. Control (**B**) Image from zymographic profiles of MMP-9 and MMP-2 activities on cells HT-29 medium.

Cell migration and invasion properties are often related with gelatinolytic activity related to increased MMP expression and activity [30]. So we also performed an MIC test for MMP-9 activity, in order to understand if the effects identified in the wound-closure assay were related to MMP-9 inhibition (Figure 13A) and a gelatinolytic assay where the MMP-2 is also included (Figure 13B). The results show a reduction in the total gelatinolytic activity with an MIC of 371.4 mg/L.

The MMP-9 activity tested in the presence of different PCs concentrations indicates that there was no significant inhibition of MMP-9 activity relative to the control.

This observation was further confirmed by the gelatine zymography assay in which the individual MMP-2 and MMP-9 activity was not significantly inhibited.

## 4. Discussion

Mentha species (*Mentha* spp.) are well-known due to their medicinal and commercial use. Indeed, the aerial parts of *Mentha* spp. are regularly used as an aromatic herb in food, tea, cosmetic and perfumery products [31,32]. Scientific research on herbal medicines in terms of their pharmacological and toxicological profiles is scarce [31,32,33]. It is worth mentioning that there is only a small number of studies focused on the mutagenic activity and toxicity of some *Mentha* spp. at high concentrations, which further supports the need for further research [31,32]. In this study, a relatively low concentration of spearmint extract on a dose of 15 mg/kg was used, which is in line with other previous studies [34,35]. Also, over recent years, our group has been evaluating the beneficial effects of different phenolic extracts in several models of inflammation, and the dose of 15 mg/kg of phenolic acids has generated consistent results and is also within the range of possibility for clinical translation [28,36,37] and use, considering a human adult of 70 kg. It is however acknowledged that further dose-response characterisation is needed following these encouraging results. Given that the pharmacological effect and the toxicity of a plant are affected by the type of compounds present [31,32], this study also sought to investigate the phenolic composition of the spearmint extract used. This revealed that hydroxycinnamic acids were the more abundant PCs (39.3% relative chromatographic area), with predominance of RA, whose concentration was found to be 1.4 mg/mL, as determined by co-injection of chromatographic standards. Aqueous extracts of different *Mentha* species presented RA concentrations of 0.66 to 12.5 mg/g of dry extract [23,38]. RA was found at concentrations of 7.1 and 26 mg/g in leaves from different *Mentha spicata* L. varieties [39,40]. Other functional groups detected at relevant concentrations in the mint extract used in this work include hydroxybenzoic acids, flavanones, luteolin glycosides and flavanols. Flavones and derivatives may also be present but at low concentrations that do not allow their unambiguous functional identification. Flavones and phenolic acids and the corresponding sugar derivatives are among the most abundant phenolics in aromatic herbs [41]. RA and several hydroxy and metoxiflavones have been identified in a methanol extract of *Mentha spicata* [42]. Linarin and naringenin as well as caffeic, chlorogenic and rosmarinic acids were found in a range of concentrations of 0.16 to 5 mg/g DW in Australian native mints [43]. Flavones and flavanones glycosides were also identified as the major phenolic compounds of peppermint tea (*Mentha piperita* L.) [44].

The phenolic composition of the spearmint used in this study is in agreement with the findings of other authors in prior studies, presenting the same compounds in their composition [42,43]. Flavones, phenolic acids and the corresponding glycosylated derivatives are the most abundant PCs in aromatic herbs [41]. When comparing the results obtained with multiple studies of various aqueous extracts of different Mentha species, all presented RA in their composition (0.66 to 12.5 mg/g of dry extract) [38,45]. RA presents several pharmacological and biological activities, including anti-inflammatory, anti-oxidant, anti-apoptotic and anti-mutagenic activities [46]. A previous work published by our group reported that RA was the main constituent responsible for the anti-inflammatory activity exhibited by a phenolic rosemary extract (*Rosmarinus officinalis*) as evidenced by a marked attenuation of paw oedema in rats paw oedema induced with carrageenan. In the same study, RA was demonstrated to be able to reduce liver injury induced via hepatic ischemia-reperfusion and it also reduced organ injury in a multi-organ dysfunction syndrome induced by burn injury [18]. Additionally, in a study where three different doses of RA were administered to animals subjected to an experimental model of IBD, RA has been shown to stop the activation of nuclear factor-kappa B (NF-κB) and signal transducer and activator of transcription 3 (STAT-3) and, afterwards, reduce the action of pro-survival genes that are regulated by these transcription factors [47]. 

Given this data, our group hypothesised that the administration of this extract to animals would be beneficial in experimental inflammation models, and proceeded to screen its anti-oxidant and anti-inflammatory effects with the aim of evaluating its potential efficacy in attenuation of inflammatory events, from acute to chronic inflammation. For that purpose, this study used the experimental paw oedema model and experimental colitis model, given that insistent inflammatory responses may lead to chronic inflammation, which sets up an extended pathological condition. Chronic inflammation has adverse cellular effects, largely through the extreme production of free radicals and a reduction in antioxidants, which may eventually lead to further expressions of chronic non-hereditary diseases like cancer, cardiovascular diseases, autoimmune diseases, degenerative processes intrinsic to aging, amongst others [48,49]. In parallel, the potential to interfere with some early signalling in colorectal cancer cells activity given the connection between chronic inflammation and the origins of cancer was tested. As colitis-associated colorectal cancer is an example of cancer that develops in circumstance of chronic inflammation, the in vitro cancer model that employs colon adenocarcinoma cells, HT-29 was tested [8].

Given that there is a wide variety of antioxidant assays, different assays must be performed to guarantee better evaluation of results, display a broader variety of possible uses and demonstrate the antioxidant capacity of an extract [50]. In the FRAP assay, spearmint showed a notable ferric ion reducing activities with a mean value of 25.86 ± 0.77 μmol Fe^2+^/mL and 333.00 ± 9.86 μmol Fe^2+^/g of dried leaves. The CUPRAC assay result is really important since it gives a precise estimation of the proper antioxidant capability of intricate samples [51]. The spearmint also showed a significant antioxidant effect in CUPRAC assay with a mean value of 37.47 ± 0.67 µmol AAE/mL and 482.50 ± 8.60 µmol AAE/g of dried leaves. The DPPH radical-scavenging assay was also performed and presented a mean value of 2.64 ± 0.03 mg AAE/mL and 33.96 ± 0.32 mg of AAE/g of dried leaves. The superoxide anion radical scavenging presented a mean result of 56.54 ± 5.48 μmol GAE/mL and 728.00 ± 70.60 μmol GAE/g of dried leaves.

In all these assays, the spearmint extract demonstrated antioxidant capacity, further suggesting that spearmint is enriched with natural antioxidants. This anti-oxidant activity is crucial to the management of IBD, because reactive oxygen species (ROS), inflammation, and increased expression of inflammatory chemokines/cytokines, report back-and-forth in the pathogenesis of ulcerative colitis [52]. Furthermore, oxidative stress and inflammation are also powerfully connected with colon carcinogenesis [53]. Change in the expression form of Nrf-2 and NF-κB has been described in UC in which functional crosstalk among these two crucial paths has been proposed [54]. In previous studies, experimental colitis has been ameliorated with up-regulation of Nrf-2 gene expression, suggesting these pathways could be potential pharmacological targets for the handling of IBD [53,54]. These results are also in accordance with preceding works that verified that ethanolic extracts of *Mentha spicata* L. exhibit a significant antioxidant capacity [55,56]. 

Furthermore, a number of reviews have focused on the valuable properties of antioxidants and polyphenols in the handling of several autoimmune diseases, including IBD and suggesting even that antioxidants might represent a significant part in the prevention of CRC [57,58,59,60,61,62,63]. Many of the mechanisms proposed as being responsible for these beneficial effects are related to the direct effect in reducing oxidative stress, but a role of direct inhibition of several inflammatory pathways has also been suggested [57,58,60,61].

The paw oedema analysis was mainly done to assess the acute anti-inflammatory action of the spearmint. In comparison to the sham group, sub plantar injection of carrageenan in rats led to a noteworthy increase of paw volume after 6 h. Paw oedema volume in the group treated with spearmint was reduced by 36%, when compared to the carrageenan group. This data is in accordance with preceding works from this team, where the administration of extracts with an enriched phenolic fraction, at the same animal dose levels, presented similar results on the paw oedema assay [36]. With regard to the positive controls (indomethacin, trolox and tempol) their results show a similar effect in comparison with spearmint. Indomethacin is a first-generation non-steroid inflammatory drug that acts through a non-selective inhibition of both COX-1 and COX-2 isozymes [64]. Trolox, a water-soluble vitamin E similar, has been used as a positive control in trolox equivalent antioxidant capacity and oxygen radical antioxidant capacity analyses due to its high antioxidative effect [65]. Finally, tempol is known as a potent antioxidant, it acts as a membrane-permeable radical scavenger, mainly inhibiting the mechanism involved in the production of superoxide anion, consequently contributing to decreasing oxidative stress and has been shown to exhibit anti-inflammatory effects in few experimental models [66,67]. Additionally, it has been shown to modulate an important pathway that controls the deleterious effect of oxidative stress in tissues that are mediated by the transcription factor Nrf2 [68], a pathway also known to play a role in UC [53,54]. 

Our results show that spearmint has a significant anti-inflammatory effect, as evidenced by the statistically significant reduction of the volume of paw oedema. Furthermore, the results obtained after spearmint administration were similar to those obtained with positive controls (known anti-oxidant and anti-inflammatory substances), which in turn can suggest that spearmint may have, at least in part, similar mechanisms of action as indomethacin, trolox and tempol. 

This study also showed that the spearmint extract had a beneficial effect on experimental colitis, since the colon lesions were significantly decreased, contributing to a reduction in mortality. According to previous studies, mice treated with other antioxidant substances presented these beneficial effects, including the decreased mortality rate [34,47,69,70].

Evaluation of COX-2 and iNOS activation in the colitis model after immunostaining of gut tissue for COX-2 and iNOS showed that the colitis group exhibits marked production of COX-2 and iNOS, which is in accordance with previous studies, given the inflammation associated to colitis in these animals [28]. Colon tissue from treated animals with spearmint extract exhibited high expression of COX-2 but no iNOS.

COX-2 and iNOS are believed to be connected with IBD pathology, because their expression is increased during the inflammatory process associated with the disease [6,71]. As we could demonstrate in the carrageenan-induced paw oedema assay, spearmint administration had a similar result to those obtained with known anti-inflammatory substances, suggesting that spearmint may share some of the same mechanisms of action, except for indomethacin a COX-1 and COX-2 inhibitor. This data suggests that spearmint administration may reduce the inflammation associated with colitis, at least in part, by the decrease of oxidative stress. Surprisingly, in this experimental model of colitis, the beneficial effect of spearmint extract appears to be unrelated to inhibition of COX-2 expression. Although it is known that COX-2 is a pivotal enzyme in inflammatory processes, including in the colon, we can suggest that spearmint extract exerts its anti-inflammatory effects through other mechanisms. Studies have shown that dietary polyphenols were able to inhibit several transcription factors known to induce both COX-2 and iNOS expression, like NF-κB, JAK/STAT and MAPK, in general processes of inflammation and immunomodulation [12], intestinal inflammation [45] and CRC [46]. Given the complex network of different cellular pathways and mediators that regulate the inflammatory process we can hypothesise that spearmint extract acts through antioxidant and anti-inflammatory effects that, taking into consideration the variety of over- and under-regulation of pathways involved, the pleiotropic effect leads to an attenuation of injury (both in local inflammation in the paw and in colitis) and an overall beneficial effect.

One of the mechanisms that we identified as a possible target for spearmint extract modulation is iNOS expression. In this study, iNOS expression was significantly attenuated by spearmint extract administration that might have played a crucial role in the attenuation of the anti-inflammatory process, which is in accordance with previously published data on spearmint effects [47,72] with previous publications from this team’s work group regarding the effects of phenolic extracts in this model of experimental colitis [28]. It is also in line with publications regarding the effect of RA in experimental models of IBD, the phenolic compound identified as the main constituent of this spearmint extract [73].

In a previous publication regarding the study of the beneficial effects of RA in experimental colitis, the administration of RA (25, 50 and 100 mg/kg p.o., three days prior to colitis induction and then daily for eight days) significantly attenuated disease severity and the histological analysed score of colons in DSS-induced colitis in mice [73]. Especially for the higher dose, RA administration was seen to decrease nitric oxide (NO) production and, among other inflammatory mediators, also reduced the expression of iNOS. Inhibition of NO production can be directly correlated to reduction of iNOS expression and is an important target in inflammatory processes since NO is an important initiator of oxidant and pro-inflammatory pathways. Its role in human IBD has also been investigated and studies have demonstrated that the level of NO production by iNOS exhibits a high correlation with the intensity of disease in IBD [74] and was in fact proposed as a new and useful biomarker in the clinical setting, namely in diagnostic protocols and also in the monitoring of patients with colitis or CD [75].

Spearmint extract administration was therefore capable of attenuating the harshness of experimental colitis, as evidenced by the beneficial effect in macroscopic signs of colon injury, histologic markers and iNOS expression. These results might suggest that this beneficial result can be related to the antioxidant activity and anti-inflammatory effects of this phenolic extract, as showed by the results of the antioxidant and in vivo inflammation study.

It is widely known that patients diagnosed with UC present a higher risk of developing CRC, with statistics showing that the risk may be 2–3 times higher compared to the general population [76,77,78]. Tumour initiation and progression processes are dependent of multiple factors, especially regarding the influence of the environment and diet, with the latter being able to either ameliorate or even increase CRC risk [79]. Chronic activation of inflammatory signalling pathways that lead to a rise of ROS creation by the colon are considered to be the primary contributing factor bridging IBD into CRC [7,80,81]. 

Spearmint extract inhibited more than 50% the migration/proliferation of HT-29 cells into the wound made in the cell monolayer in the wound healing assay and the MIC assay with these cells determined that spearmint extract inhibited their proliferation more than 50% for concentrations less than 600 μg/mL. Gelatinolytic assays were then handled to find out if spearmint extract could inhibit MMP-9 and MMP-2 activities, and if these could be related to earlier results found in the in vivo and in vitro models. MMP-2 and MMP-9 activity was not significantly inhibited in the presence of spearmint extract.

Although it appears that inhibition of MMPs would be beneficial to improve signs and symptoms of IBD and to prevent the development of CRC [82], previous studies from this team’s work have already shown that phenolic extracts might reduce inflammation in experimental colitis and reduce migration/invasion and proliferation capacities of HT-29 cells without acting through the reduction of MMPs activities [28], also suggesting that these spearmint extract effects are not related with MMP’s inhibition but with other mechanisms. In fact, nitric oxide production and iNOS expression have been identified as having pivotal roles in the proliferation and metastasis of several cancer types [83,84], including CRC [85], which might be a potential target explaining the beneficial effects of this spearmint extract. 

## 5. Conclusions

*Mentha* spp. is frequently ingested as an aromatic herb in food and tea infusions. Phenolic compounds are the major components of spearmint extract and given the relevance of RA in inflammatory processes, its high concentration in the extract might be one of the main reasons for the beneficial effects observed. Preliminary screening of the extracts’ antioxidant and anti-inflammatory properties suggested that it might be beneficial in an experimental model of IBD. Indeed, our findings showed that the administration of a spearmint phenolic extract attenuated the severity of inflammation associated with experimental colitis, including a decrease of the mortality rate of 15.3%. The inhibition of iNOS expression appears to be relevant for the effects observed with the spearmint extract, which may reduce the inflammation associated to colitis along with a possible pleiotropic effect in other inflammatory and oxidative pathways. The mechanisms involved in the impairment of the inflammatory process in this experimental model of colitis may also be responsible for the inhibitory effects exhibited by the extract in the migration/invasion properties of HT-29 colorectal cancer cells. 

Studies focusing on the characterization of the chemical composition of several spearmint infusions obtained by different methods and spearmint strains have shown that the intake of RA by this route (equivalent to three cups/300 mL every day) would range between 50 and 560 mg/day [86,87], which is half of the dose obtained with a single administration (980 mg) of the extract when translated to a 70 kg adult. Therefore, this extract yielded a high concentration of RA, thus being a good candidate for further research and development of formulations intended to provide a dose of RA and other phenolic compounds with potential complementary therapeutic effects.

Given the role of inflammatory processes in the progression of colorectal cancer and the important link between inflammation and cancer, spearmint extract might be a useful pharmacological tool for the adjuvant management of IBD patients and may open up new research opportunities in the impairment of colon cancer progression. 

## Figures and Tables

**Table 1:** Classification of diarrhoea harshness.

Score	Faeces Consistency
0	Normal (firm pellets)
1	A little mucous
2	Smooth
3	Watery

**Table 2:** Spearmint extract antioxidant activity.

Antioxidant Test	Mint Extract	Units
**FRAP**	25.86 ± 0.77	µmol Fe^2+^/mL
	333.00 ± 9.86	µmol Fe^2+^/g dry plant
**CUPRAC**	2.38 ± 0.06	µmol EAA/mL
	482.50 ± 8.60	µmol EAA/g dry plant
**DPPH**	2.637 ± 0.025	mg EAA/mL
	33.96 ± 0.32	mg de EAA/g dry plant
**Superoxide anion radical scavenging**	56.54 ± 5.48	µmol EAG/mL
	728.0 ± 70.6	µmol EAG/g dry plant

**Table 3:** Effect of spearmint treatment in the evaluated macroscopic markers of TNBS-induced colitis.

Group	Colon Length (cm)	Lesion Extension (cm)	Diarrhoea Score	Mortality (%)
**Sham**	14.5 ± 0.2	0.0 ± 0.0	0.0 ± 0.0	0
**Ethanol (50%)**	14.1 ± 0.4	0.0 ± 0.0	0.0 ± 0.0	0
**TNBS**	11.8 ± 0.5^#^	3.6 ± 0.4^#^	3.0 ± 0.0^#^	37.5
**TNBS + Spearmint**	14.1 ± 1.2*	1.9 ± 1.0*	0.9 ± 0.8*	22.2

Legend: EtOH: Ethanol; TNBS: 2,4,6-Trinitrobenzenesulfonic acid. # *p* < 0.001 compared with ethanol group; * *p* < 0.001 compared with TNBS group.

## References

[1] Engel M.A., Neurath M.F. (2010). New pathophysiological insights and modern treatment of IBD. J. Gastroenterol..

[2] De Lange K.M., Barrett J.C. (2015). Understanding inflammatory bowel disease via immunogenetics. J. Autoimmun..

[3] Sobczak M., Fabisiak A., Murawska N., Wesołowska E., Wierzbicka P., Wlazłowski M., Wójcikowska M., Zatorski H., Zwolińska M., Fichna J. (2014). Current overview of extrinsic and intrinsic factors in etiology and progression of inflammatory bowel diseases. Pharmacol. Rep..

[4] Katz J.A., Itoh J., Fiocchi C. (1999). Pathogenesis of inflammatory bowel disease. Curr. Opin. Gastroenterol..

[5] Pithadia A.B., Jain S. (2011). Treatment of inflammatory bowel disease (IBD). Pharmacol. Rep..

[6] Wang D., DuBois R.N. (2011). The role of COX-2 in intestinal and colorectal cancer. Oncogene.

[7] Romano M., DE Francesco F., Zarantonello L., Ruffolo C., Ferraro G.A., Zanus G., Giordano A., Bassi N., Cillo U. (2016). From Inflammation to Cancer in Inflammatory Bowel Disease: Molecular Perspectives. Anticancer Res..

[8] Taleban S., Elquza E., Gower-Rousseau C., Peyrin-Biroulet L. (2016). Cancer and inflammatory bowel disease in the elderly. Dig. Liver Dis..

[9] Zhao C.Z., Wang Y., Tang F.D., Zhao X.J., Xu Q.P., Xia J.F., Zhu Y.F. (2008). Effect of spearmint oil on inflammation, oxidative alteration and Nrf2 expression in lung tissue of COPD rats. J. Zhejiang Univ. Med Sci..

[10] Vejdani R., Shalmani H.R.M., Mir-Fattahi M., Sajed-Nia F., Abdollahi M., Zali M.R., Alizadeh A.H.M., Bahari A., Amin G. (2006). The efficacy of an herbal medicine, Carmint, on the relief of abdominal pain and bloating in patients with irritable bowel syndrome: A pilot study. Dig. Dis. Sci..

[11] Tisserat B., Berhow M., Vaughn S.F. (2009). Spearmint plantlet culture system as a means to study secondary metabolism. Methods Mol. Biol..

[12] Cirlini M., Mena P., Tassotti M., Herrlinger K., Nieman K., Dall’Asta C., Del Rio D. (2016). Phenolic and Volatile Composition of a Dry Spearmint (*Mentha spicata* L.) Extract. Molecules.

[13] Yun S.Y., Hur Y.G., Sang M.A., Lee J., Ahn C., Won J. (2003). Synergistic immunosuppressive effects of rosmarinic acid and rapamycin in vitro and in vivo. Transplantation.

[14] Renzulli C., Galvano F., Pierdomenico L., Speroni E., Guerra M.C. (2004). Effects of rosmarinic acid against aflatoxin B1 and ochratoxin-a-induced cell damage in a human hepatoma cell line (Hep G2). J. Appl. Toxicol..

[15] Iuvone T. (2006). The Spice Sage and Its Active Ingredient Rosmarinic Acid Protect PC12 Cells from Amyloid-beta Peptide-Induced Neurotoxicity. J. Pharmacol. Exp. Ther..

[16] Petersen M. (2003). Rosmarinic acid. Phytochemistry.

[17] Lasrado J.A., Nieman K.M., Fonseca B.A., Sanoshy K.D., Schild A.L., Herrlinger K.A. (2017). Safety and tolerability of a dried aqueous spearmint extract. Regul. Toxicol. Pharmacol..

[18] Rocha J., Eduardo-Figueira M., Barateiro A., Fernandes A., Brites D., Bronze R., Duarte C.M., Serra A.T., Pinto R., Freitas M. (2015). Anti-inflammatory Effect of Rosmarinic Acid and an Extract of Rosmarinus officinalis in Rat Models of Local and Systemic Inflammation. Basic Clin. Pharmacol. Toxicol..

[19] Mizuno R., Kawada K., Itatani Y., Ogawa R., Kiyasu Y., Sakai Y. (2019). The Role of Tumor-Associated Neutrophils in Colorectal Cancer. Int. J. Mol. Sci..

[20] Mateus V., Rocha J., Alves P., Mota-Filipe H., Sepodes B., Pinto R.M.A. (2017). Anti-Inflammatory Effect of Erythropoietin in the TNBS-induced Colitis. Basic Clin. Pharmacol. Toxicol..

[21] Shacter E., Weitzman S.A. (2002). Chronic inflammation and cancer. Oncology.

[22] Khatami M. (2011). Unresolved inflammation: ‘immune tsunami’ or erosion of integrity in immune-privileged and immune-responsive tissues and acute and chronic inflammatory diseases or cancer. Expert Opin. Biol. Ther..

[23] Koşar M., Göger F., Can Başer K.H. (2008). In Vitro Antioxidant Properties and Phenolic Composition of Salvia virgata Jacq. from Turkey. J. Agric. Food Chem..

[24] Ramful D., Bahorun T., Bourdon E., Tarnus E., Aruoma O.I. (2010). Bioactive phenolics and antioxidant propensity of flavedo extracts of Mauritian citrus fruits: Potential prophylactic ingredients for functional foods application. Toxicology.

[25] Apak R., Güçlü K., Ozyürek M., Karademir S.E. (2004). Novel total antioxidant capacity index for dietary polyphenols and vitamins C and E, using their cupric ion reducing capability in the presence of neocuproine: CUPRAC method. J. Agric. Food Chem..

[26] Miceli N., Trovato A., Dugo P., Cacciola F., Donato P., Marino A., Bellinghieri V., La Barbera T.M., Gljvenç A., Taviano M.F. (2009). Comparative analysis of flavonoid profile, antioxidant and antimicrobial activity of the berries of Juniperus communis L. var. communis and Juniperus communis L. var. saxatilis Pall, from Turkey. J. Agric. Food Chem..

[27] Valentão P., Fernandes E., Carvalho F., Andrade P.B., Seabra R.M., Bastos M.L. (2001). Antioxidant activity of Centaurium erythraea infusion evidenced by its superoxide radical scavenging and xanthine oxidase inhibitory activity. J. Agric. Food Chem..

[28] Direito R., Lima A., Rocha J., Ferreira R.B., Mota J., Rebelo P., Fernandes A., Pinto R., Alves P., Bronze R. (2017). Dyospiros kaki phenolics inhibit colitis and colon cancer cell proliferation, but not gelatinase activities. J. Nutr. Biochem..

[29] Lima A.I.G., Mota J., Monteiro S.A.V.S., Ferreira R.M.S.B. (2016). Legume seeds and colorectal cancer revisited: Protease inhibitors reduce MMP-9 activity and colon cancer cell migration. Food Chem..

[30] Quaranta V. (2000). Cell Migration through Extracellular Matrix. J. Cell Biol..

[31] Ulbricht C., Costa D., M Grimes Serrano J., Guilford J., Isaac R., Seamon E., Varghese M. (2010). An Evidence-Based Systematic Review of Spearmint by the Natural Standard Research Collaboration. J. Diet. Suppl..

[32] Daneshbakhsh D., Asgarpanah J., Najafizadeh P., Rastegar T., Mousavi Z. (2018). Safety Assessment of Mentha mozaffarianii Essential Oil: Acute and Repeated Toxicity Studies. Iran. J. Med. Sci..

[33] Calixto J.B. (2000). Efficacy, safety, quality control, marketing and regulatory guidelines for herbal medicines (phytotherapeutic agents). Braz. J. Med. Biol. Res. Rev. Bras. Pesqui. Med. Biol..

[34] Jagetia G.C., Baliga M.S. (2002). Influence of the leaf extract of Mentha arvensis Linn. (mint) on the survival of mice exposed to different doses of gamma radiation. Strahlenther. Onkol..

[35] Bastaki S.M., Adeghate E., Amir N., Ojha S., Oz M. (2018). Menthol inhibits oxidative stress and inflammation in acetic acid-induced colitis in rat colonic mucosa. Am. J. Transl. Res..

[36] Figueira M.E., Câmara M.B., Direito R., Rocha J., Serra A.T., Duarte C.M.M., Fernandes A., Freitas M., Fernandes E., Marques M.C. (2014). Chemical characterization of a red raspberry fruit extract and evaluation of its pharmacological effects in experimental models of acute inflammation and collagen-induced arthritis. Food Funct..

[37] Figueira M.-E., Oliveira M., Direito R., Rocha J., Alves P., Serra A.-T., Duarte C., Bronze R., Fernandes A., Brites D. (2016). Protective effects of a blueberry extract in acute inflammation and collagen-induced arthritis in the rat. Biomed. Pharmacother..

[38] Fecka I., Turek S. (2008). Determination of polyphenolic compounds in commercial herbal drugs and spices from Lamiaceae: Thyme, wild thyme and sweet marjoram by chromatographic techniques. Food Chem..

[39] Wang H., Provan G.J., Helliwell K. (2004). Determination of rosmarinic acid and caffeic acid in aromatic herbs by HPLC. Food Chem..

[40] Narasimhamoorthy B., Zhao L.Q., Liu X., Yang W., Greaves J.A. (2015). Differences in the chemotype of two native spearmint clonal lines selected for rosmarinic acid accumulation in comparison to commercially grown native spearmint. Ind. Crops Prod..

[41] Costa D.C., Costa H.S., Albuquerque T.G., Ramos F., Castilho M.C., Sanches-Silva A. (2015). Advances in phenolic compounds analysis of aromatic plants and their potential applications. Trends Food Sci. Technol..

[42] Yamamura S., Ozawa K., Ohtani K., Kasai R., Yamasaki K. (1998). Antihistaminic flavones and aliphatic glycosides from *Mentha spicata*. Phytochemistry.

[43] Hanafy D.M., Prenzler P.D., Burrows G.E., Ryan D., Nielsen S., El Sawi S.A., El Alfy T.S., Abdelrahman E.H., Obied H.K. (2017). Biophenols of mints: Antioxidant, acetylcholinesterase, butyrylcholinesterase and histone deacetylase inhibition activities targeting Alzheimer’s disease treatment. J. Funct. Foods.

[44] Kogiannou D.A.A., Kalogeropoulos N., Kefalas P., Polissiou M.G., Kaliora A.C. (2013). Herbal infusions; their phenolic profile, antioxidant and anti-inflammatory effects in HT29 and PC3 cells. Food Chem. Toxicol..

[45] Koşar M., Dorman H.J.D., Can Başer K.H., Hiltunen R. (2004). Screening of Free Radical Scavenging Compounds in Water Extracts of *Mentha* Samples Using a Postcolumn Derivatization Method. J. Agric. Food Chem..

[46] Ahmed H. (2018). Ethnomedicinal, Phytochemical and Pharmacological Investigations of Perilla frutescens (L.) Britt. Molecules.

[47] Jin B., Chung K., Se-yun C., Lee M., Hwang S., Noh S. (2017). Rosmarinic acid suppresses colonic inflammation in dextran sulphate sodium (DSS)-induced mice via dual inhibition of NF-κB and STAT3 activation. Sci. Rep..

[48] Medzhitov R. (2010). Inflammation 2010: New adventures of an old flame. Cell.

[49] Rubin D.C., Shaker A., Levin M.S. (2012). Chronic intestinal inflammation: Inflammatory bowel disease and colitis-associated colon cancer. Front. Immunol..

[50] Kanatt S.R., Chander R., Sharma A. (2007). Food Chemistry Antioxidant potential of mint (*Mentha spicata* L.) in radiation-processed lamb meat. Food Chem..

[51] Ozyurt D., Demirata B., Apak R. (2011). Determination of Total Antioxidant Capacity by a New Spectrofluorometric Method Based on Ce(IV) Reduction: Ce(III) Fluorescence Probe for CERAC Assay. J. Fluoresc..

[52] Khodir A.E., Said E., Atif H., ElKashef H.A., Salem H.A. (2019). Targeting Nrf2/HO-1 signaling by crocin: Role in attenuation of AA-induced ulcerative colitis in rats. Biomed. Pharmacother..

[53] Fang R., Wu R., Zuo Q., Yin R., Zhang C., Wang C., Guo Y., Yang A.Y., Li W., Lin L. (2018). Sophora flavescens Containing-QYJD Formula Activates Nrf2 Anti-Oxidant Response, Blocks Cellular Transformation and Protects Against DSS-Induced Colitis in Mouse Model. Am. J. Chin. Med..

[54] Saber S., Khalil R.M., Abdo W.S., Nassif D., El-Ahwany E. (2019). Olmesartan ameliorates chemically-induced ulcerative colitis in rats via modulating NFκB and Nrf-2/HO-1 signaling crosstalk. Toxicol. Appl. Pharmacol..

[55] Mata A.T., Proença C., Ferreira A.R., Serralheiro M.L.M., Nogueira J.M.F., Araújo M.E.M. (2007). Antioxidant and antiacetylcholinesterase activities of five plants used as Portuguese food spices. Food Chem..

[56] Fatiha B., Didier H., Naima G., Khodir M., Martin K., Léocadie K., Caroline S., Mohamed C., Pierre D. (2015). Phenolic composition, in vitro antioxidant effects and tyrosinase inhibitory activity of three Algerian *Mentha* species: *M. spicata* (L.), *M. pulegium* (L.) and *M. rotundifolia* (L.) Huds (Lamiaceae). Ind. Crops Prod..

[57] Gothai S., Muniandy K., Gnanaraj C., Ibrahim I.A.A., Shahzad N., Al-Ghamdi S.S., Ayoub N., Veeraraghavan V.P., Kumar S.S., Esa N.M. (2018). Pharmacological insights into antioxidants against colorectal cancer: A detailed review of the possible mechanisms. Biomed. Pharmacother..

[58] Khan H., Sureda A., Belwal T., Çetinkaya S., Süntar İ., Tejada S., Devkota H.P., Ullah H., Aschner M. (2019). Polyphenols in the treatment of autoimmune diseases. Autoimmun. Rev..

[59] Peng J., Zheng T.-T., Li X., Liang Y., Wang L.-J., Huang Y.-C., Xiao H.-T. (2019). Plant-Derived Alkaloids: The Promising Disease-Modifying Agents for Inflammatory Bowel Disease. Front. Pharmacol..

[60] Barbalho S.M., Bosso H., Salzedas-Pescinini L.M., de Alvares Goulart R. (2019). Green tea: A possibility in the therapeutic approach of inflammatory bowel diseases?. Complement. Ther. Med..

[61] Ribeiro D., Proenca C., Rocha S., Lima J.L.F.C., Carvalho F., Fernandes E., Freitas M. (2018). Immunomodulatory Effects of Flavonoids in the Prophylaxis and Treatment of Inflammatory Bowel Diseases: A Comprehensive Review. Curr. Med. Chem..

[62] Nunes S., Danesi F., Del Rio D., Silva P. (2018). Resveratrol and inflammatory bowel disease: The evidence so far. Nutr. Res. Rev..

[63] Salaritabar A., Darvishi B., Hadjiakhoondi F., Manayi A., Sureda A., Nabavi S.F., Fitzpatrick L.R., Nabavi S.M., Bishayee A. (2017). Therapeutic potential of flavonoids in inflammatory bowel disease: A comprehensive review. World J. Gastroenterol..

[64] Abdelall E.K.A., Lamie P.F., Ahmed A.K.M., El-Nahass E.-S. (2019). COX-1/COX-2 inhibition assays and histopathological study of the new designed anti-inflammatory agent with a pyrazolopyrimidine core. Bioorg. Chem..

[65] Wada M., Wada M., Ikeda R., Fuchigami Y., Koyama H., Ohkawara S., Kawakami S., Kuroda N., Nakashima K. (2016). Quantitative and antioxidative behavior of Trolox in rats’ blood and brain by HPLC-UV and SMFIA-CL methods. Luminescence.

[66] Sepodes B., Maio R., Pinto R., Marques C., Mendes-do-Vale J., McDonald M., Thiemermann C., Mota-Filipe H. (2004). Tempol, an intracelullar free radical scavenger, reduces liver injury in hepatic ischemia-reperfusion in the rat. Transplant. Proc..

[67] Thiemermann C. (2003). Membrane-permeable radical scavengers (tempol) for shock, ischemia-reperfusion injury, and inflammation. Crit. Care Med..

[68] Bernardy C.C.F., Zarpelon A.C., Pinho-Ribeiro F.A., Calixto-Campos C., Carvalho T.T., Fattori V., Borghi S.M., Casagrande R., Verri W.A. (2017). Tempol, a Superoxide Dismutase Mimetic Agent, Inhibits Superoxide Anion-Induced Inflammatory Pain in Mice. Biomed Res. Int..

[69] Koutroumanidou E., Kimbaris A., Kortsaris A., Bezirtzoglou E., Polissiou M., Charalabopoulos K., Pagonopoulou O. (2013). Increased seizure latency and decreased severity of pentylenetetrazol-induced seizures in mice after essential oil administration. Epilepsy Res. Treat..

[70] Michelucci A., Paolini C., Canato M., Wei-Lapierre L., Pietrangelo L., De Marco A., Reggiani C., Dirksen R.T., Protasi F. (2015). Antioxidants protect calsequestrin-1 knockout mice from halothane- and heat-induced sudden death. Anesthesiology.

[71] Kolios G., Valatas V., Ward S.G. (2004). Nitric oxide in inflammatory bowel disease: A universal messenger in an unsolved puzzle. Immunology.

[72] Ghasemzadeh Rahbardar M., Amin B., Mehri S., Mirnajafi-Zadeh S.J., Hosseinzadeh H. (2017). Anti-inflammatory effects of ethanolic extract of Rosmarinus officinalis L. and rosmarinic acid in a rat model of neuropathic pain. Biomed. Pharmacother..

[73] Zhao L., Zhang Y., Liu G., Hao S., Wang C., Wang Y. (2018). Black rice anthocyanin-rich extract and rosmarinic acid, alone and in combination, protect against DSS-induced colitis in mice. Food Funct..

[74] Cross R.K., Wilson K.T. (2003). Nitric oxide in inflammatory bowel disease. Inflamm. Bowel Dis..

[75] Lundberg J.O., Hellström P.M., Fagerhol M.K., Weitzberg E., Roseth A.G. (2005). Technology Insight: Calprotectin, lactoferrin and nitric oxide as novel markers of inflammatory bowel disease. Nat. Clin. Pract. Gastroenterol. Hepatol..

[76] Lopez A., Pouillon L., Beaugerie L., Danese S., Peyrin-Biroulet L. (2018). Colorectal cancer prevention in patients with ulcerative colitis. Best Pract. Res. Clin. Gastroenterol..

[77] Zhen Y., Luo C., Zhang H. (2018). Early detection of ulcerative colitis-associated colorectal cancer. Gastroenterol. Rep..

[78] Axelrad J.E., Lichtiger S., Yajnik V. (2016). Inflammatory bowel disease and cancer: The role of inflammation, immunosuppression, and cancer treatment. World J. Gastroenterol..

[79] Donovan M.G., Selmin O.I., Doetschman T.C., Romagnolo D.F. (2017). Mediterranean Diet: Prevention of Colorectal Cancer. Front. Nutr..

[80] Rogler G. (2014). Chronic ulcerative colitis and colorectal cancer. Cancer Lett..

[81] Mármol I., Sánchez-de-Diego C., Pradilla Dieste A., Cerrada E., Rodriguez Yoldi M.J. (2017). Colorectal Carcinoma: A General Overview and Future Perspectives in Colorectal Cancer. Int. J. Mol. Sci..

[82] Yan C., Boyd D.D. (2007). Regulation of matrix metalloproteinase gene expression. J. Cell. Physiol..

[83] Cheng H., Wang L., Mollica M., Re A.T., Wu S., Zuo L. (2014). Nitric oxide in cancer metastasis. Cancer Lett..

[84] Granados-Principal S., Liu Y., Guevara M.L., Blanco E., Choi D.S., Qian W., Patel T., Rodriguez A.A., Cusimano J., Weiss H.L. (2015). Inhibition of iNOS as a novel effective targeted therapy against triple-negative breast cancer. Breast Cancer Res..

[85] Peñarando J., López-Sánchez L.M., Mena R., Guil-Luna S., Conde F., Hernández V., Toledano M., Gudiño V., Raponi M., Billard C. (2018). A role for endothelial nitric oxide synthase in intestinal stem cell proliferation and mesenchymal colorectal cancer. BMC Biol..

[86] Connelly A.E., Tucker A.J., Tulk H., Catapang M., Chapman L., Sheikh N., Yurchenko S., Fletcher R., Kott L.S., Duncan A.M. (2014). High-rosmarinic acid spearmint tea in the management of knee osteoarthritis symptoms. J. Med. Food.

[87] Rita I., Pereira C., Barros L., Santos-Buelga C., Ferreira I.C.F.R. (2016). *Mentha spicata* L. infusions as sources of antioxidant phenolic compounds: Emerging reserve lots with special harvest requirements. Food Funct..

